# Prime editing strategies to mediate exon skipping in *DMD* gene

**DOI:** 10.3389/fmed.2023.1128557

**Published:** 2023-05-25

**Authors:** Cedric Happi Mbakam, Jeanne Roustant, Joel Rousseau, Pouire Yameogo, Yaoyao Lu, Anne Bigot, Kamel Mamchaoui, Vincent Mouly, Gabriel Lamothe, Jacques P. Tremblay

**Affiliations:** ^1^CHU de Québec Research Centre, Laval University, Québec, QC, Canada; ^2^Molecular Medicine Department, Faculty of Medicine, Laval University, Québec, QC, Canada; ^3^Biology and Heath Sciences, Polytech Angers, Angers, France; ^4^Institute of Myology, Myology Research Center, Paris, France

**Keywords:** exon skipping, prime editing, CRISPR-Cas9, Duchenne muscular dystrophy, *DMD* gene, splice site

## Abstract

Duchenne muscular dystrophy is a rare and lethal hereditary disease responsible for progressive muscle wasting due to mutations in the *DMD* gene. We used the CRISPR-Cas9 Prime editing technology to develop different strategies to correct frameshift mutations in *DMD* gene carrying the deletion of exon 52 or exons 45 to 52. With optimized epegRNAs, we were able to induce the specific substitution of the GT nucleotides of the splice donor site of exon 53 in up to 32% of HEK293T cells and 28% of patient myoblasts. We also achieved up to 44% and 29% deletion of the G nucleotide of the GT splice site of exon 53, as well as inserted 17% and 5.5% GGG between the GT splice donor site of exon 51 in HEK293T cells and human myoblasts, respectively. The modification of the splice donor site for exon 51 and exon 53 provoke their skipping and allowed exon 50 to connect to exon 53 and allowed exon 44 to connect to exon 54, respectively. These corrections restored the expression of dystrophin as demonstrated by western blot. Thus, Prime editing was used to induce specific substitutions, insertions and deletions in the splice donor sites for exons 51 and 53 to correct the frameshift mutations in *DMD* gene carrying deletions of exon 52 and exons 45 to 52, respectively.

## 1. Introduction

Duchenne muscular dystrophy (DMD) is a hereditary and neuromuscular disease demonstrating a high psychosocial impact (1–3). The disease is caused by mutations in the *DMD* gene which codes for the dystrophin protein. This leads to the reduction of dystrophin expression which causes structural and functional destabilisation of dystrophin complex associated proteins (DCAP). This in turn leads to progressive muscle damage (4). Different mutations including point mutations (26% of *DMD* mutations), duplications (10% to 15% of *DMD* mutations), and exon deletions (60% to 70% of *DMD* mutations) lead to a premature stop codon in the *DMD* gene which explains the absence of dystrophin expression in DMD patients (4, 5). The vast majority of these mutations fall in two zones called *DMD* hot spots (4, 6). The first and the most important zone spans from exon 45 to exon 55. Deletions in this zone remove a part of the central rod domain and C-terminal side of the nNOS binding site (4, 6). The second zone spans exons 2 to 19. Deletions in this zone remove all or a part of the actin binding amino terminal domain and a part of the rod domain (6). Exon deletions in the *DMD* gene, one of the biggest genes in the human genome, result in frameshift mutations and lead to premature stop codons. Correcting such mutations usually requires strategies focused on exon skipping (which consists of modifying splice sites to skip over exon(s) during the mRNA synthesis) or additional in-frame exon deletions (which consists of deleting additional exons to connect adjacent exons or to form a hybrid exon with normal spectrin like repeats) (7, 8). Interestingly, the *DMD* gene is often still capable of producing a partially functional, albeit truncated, dystrophin protein following large *DMD* intragenic deletions as observed in some Becker muscular dystrophy (BMD), a form of muscular dystrophy with mild phenotype due to in-frame mutations in *DMD* gene (9, 10). In-frame deletion in *DMD* gene also inspired the micro-dystrophin gene transfer therapeutics in which, different forms of micro-dystrophin are designed and tested for their ability to express a truncated and partially functional form of dystrophin as summarized by Davies and Guiraud (11).

Different strategies for dystrophin restoration have recently been summarized by our group (12). Emphasis was given to CRISPR-Cas9 approaches which have demonstrated a strong potential for developing permanent treatments for DMD (7, 12). The CRISPR-Cas9 nuclease induces a double-strand break in the *DMD* gene and has been widely used to develop different strategies to restore dystrophin expression. These strategies include the deletion of complete exons, the formation of hybrid exons, INDEL-derived reframing, and splice site modifications. However, the most notable impediment to these nuclease-based approaches is their association with important off-target events and high levels of INDELs (7). To solve these problems and expand their scope, CRISPR nucleases were modified to develop nickases. These nickases only cleave one strand of the DNA duplex. They were first used to develop base editing which uses a sgRNA and a deaminase attached to the Cas9 nickase. Base editing has already been used in many studies to correct DMD mutations (13–16). However, the undesired deamination in the editing windows and the transcriptome remains a key challenge with base editors. CRISPR nucleases were also used to develop Prime editing which uses a prime editing gRNA (pegRNA) and a reverse transcriptase attached to the Cas9 nickase to induce a modification to the genome (17–20). Prime editing permits any base-to-base substitutions, insertions and deletions of DNA nucleotides or sequences.

To the best of our knowledge, there are no therapeutic approaches which use Prime editing to correct *DMD* exon deletions through exon skipping. In the present study, we developed different strategies to correct frameshift caused by exon deletions in the *DMD* gene using Prime editing. These mutations included the deletion of exon 52 (Del52) and exons 45 to 52 (Del45-52). It has been shown that skipping exons 51, 53, and 45 might be beneficial for 14%, 10%, and 9% of *DMD* mutations, respectively, and for 21%, 15%, and 13% of exon deletions, respectively (5). We subsequently demonstrated that Prime editing can be used to mediate exon 51 and 53 skipping by modifying their splice donor or acceptor sites by inducing substitutions, insertions, and deletions.

## 2. Methods

Some methods used in the present manuscript were previously described (21).

### 2.1. Strategies to correct the frameshift in Del52

**Figure 1:**
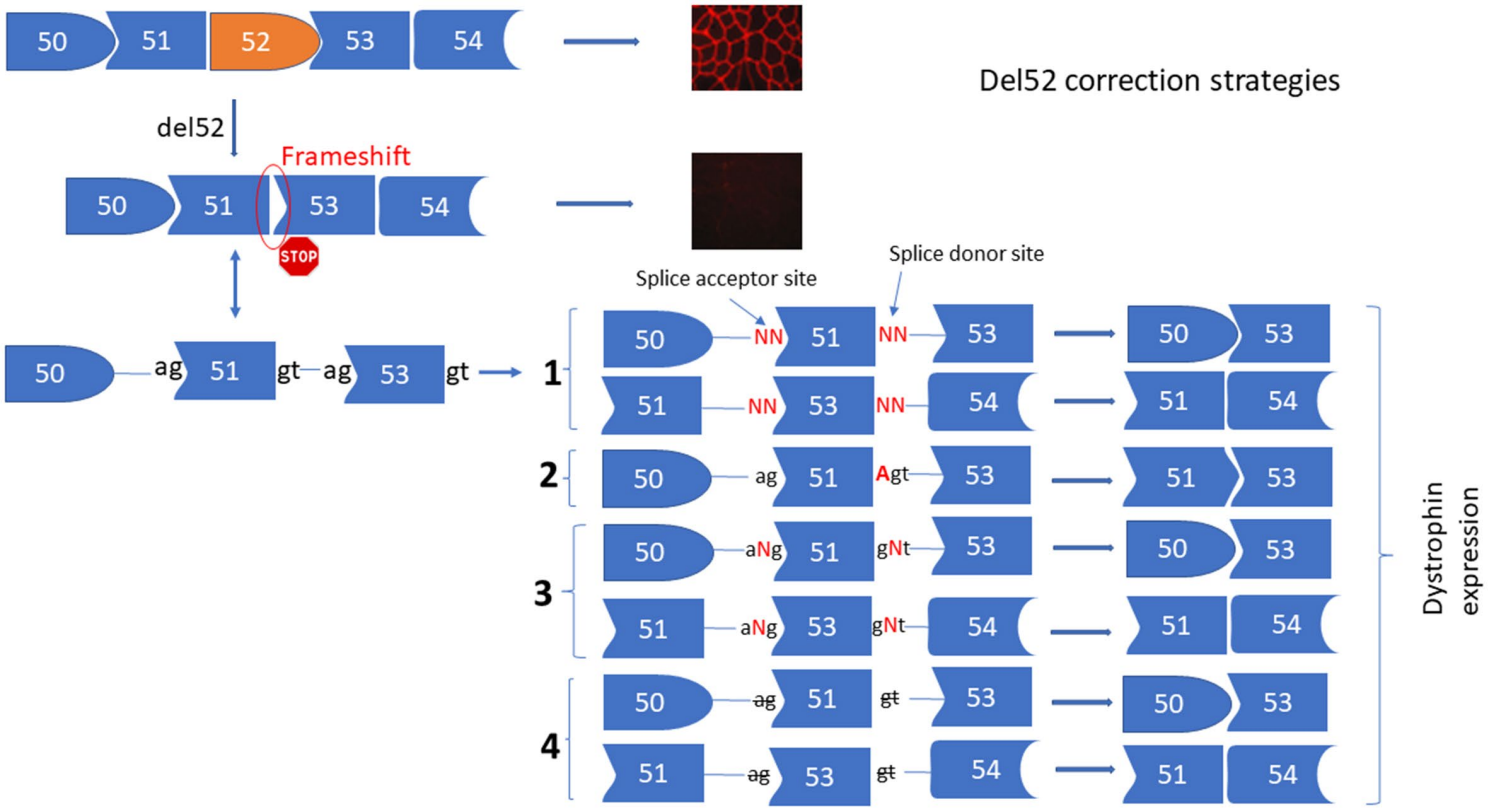
Strategies for the correction of Del52 mutation in DMD gene. This figure represents the substitution, insertion, and deletion strategies to restore the dystrophin expression for the Del52 mutation in the DMD gene using the Prime editing technology. **(1)** Shows the substitution of one or two nucleotides of the splice sites, **(2)** shows the insertion of A at the end of exon 51 or at the beginning of exon 53, or the insertion of one or many nucleotides between the two nucleotides of the splice sites **(3)** and **(4)** shows the deletion of one or many nucleotides at the splice sites.

The deletion of exon 52 (Del52) provokes a frameshift mutation resulting in a premature stop codon in exon 53. To correct Del52 we designed four different strategies to mediate the restoration of dystrophin (Figure 1). These included: (1) Substituting one or two nucleotides at the donor or the acceptor splice sites of exon 51 or exon 53 to mediate the skipping of exon 51 linking exon 50 to 53 or the skipping of exon 53 connecting exon 51 to 54. (2) Inserting an adenine (A) nucleotide at the end of exon 51 or the beginning of exon 53 to reframe the *DMD* gene by restoring the isoleucine (ATT) amino acid that has been altered by the deletion of exon 52. (3) Inserting of one or many nucleotides between the splice donor (5’GT) or acceptor (5’AG) nucleotides of exon 51 or 53 to mediate their skipping. This strategy connects exon 50 to 53 or exon 51 to 54. (4) Deleting the splice site nucleotides with or without adjacent nucleotides in the intron sequences of exon 51 or 53 to mediate their skipping.

### 2.2. Strategies to correct the frameshift in Del45-52

**Figure 2:**
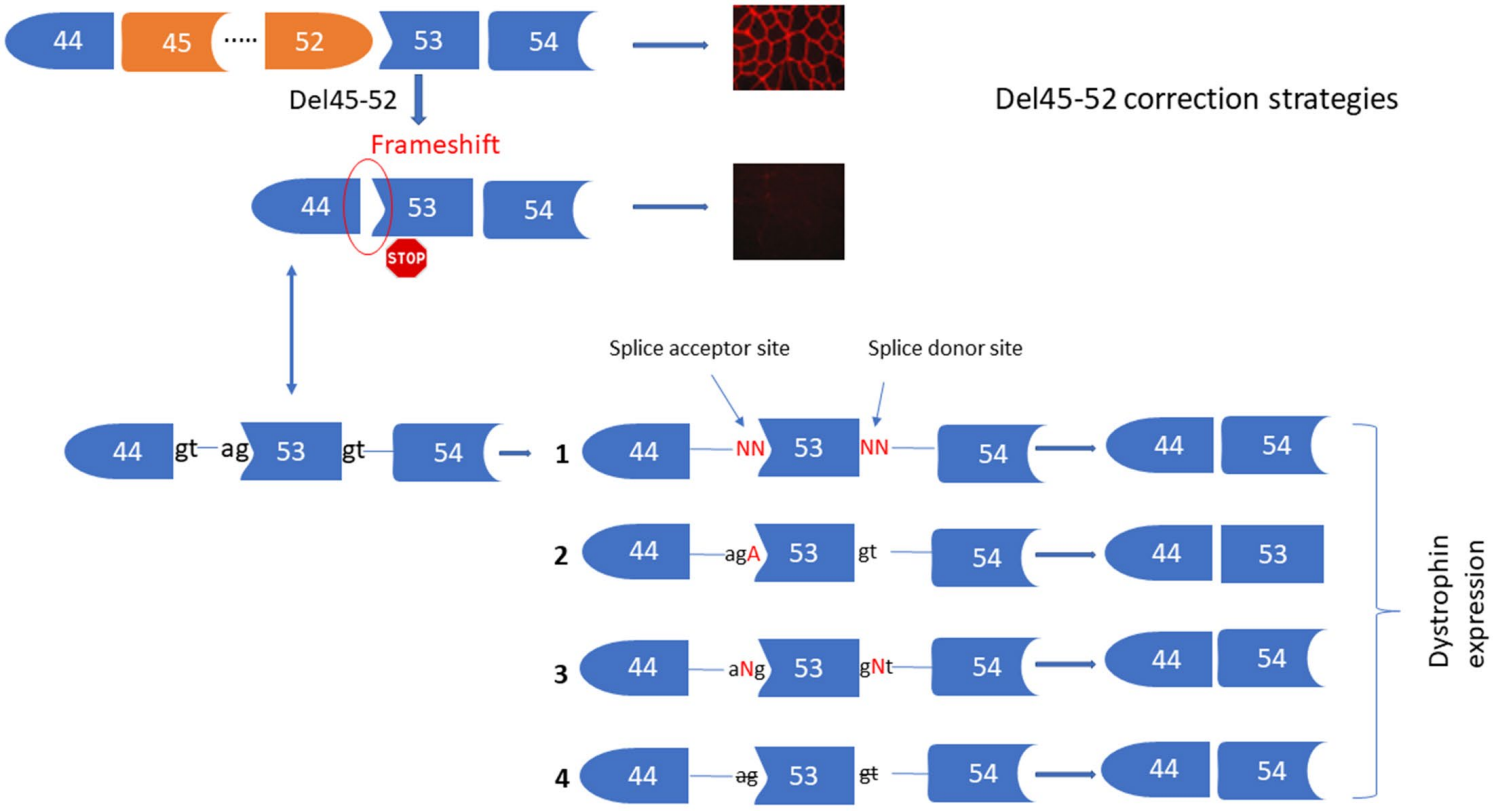
Strategies for the correction of Del45-52. This figure represents the Prime editing strategies for the corrections of Del45-52 in the *DMD* gene to mediate the dystrophin restoration. These includes the substitutions of one or two nucleotides of the splice sites 53 **(1)**, insertion of A at the end of exon 44 or the beginning of exon 53 **(2)**, insertion of one or many nucleotides between the two nucleotides of the splice sites of exon 53 **(3)**, deletion of one or many nucleotides at the splice sites of exon 53 **(4)**.

The deletion of exons 45 to 52 (Del45-52) in the *DMD* gene leads to a frameshift in exon 53 resulting in the formation of a premature stop codon and absence of the dystrophin expression under the sarcolemma. We proposed four different strategies for the correction of this mutation (Figure 2) including: (1) Substituting nucleotides at the splice donor and acceptor sites of exon 53 to mediate its skipping by linking exon 44 to exon 54. (2) Inserting an Adenine at the end of exon 44 or at the beginning of exon 54 to restore the Isoleucine codon (ATT) (i.e., the Adenine nucleotide (A) of the ATT codon was lost during the deletion of exons 45 to 52). (3) Inserting one or many nucleotides between the splice donor (5’GT) or acceptor (5’AG) nucleotides of exon 53 to mediate its skipping, connecting exon 44 to 54. (4) Deleting only the splice site nucleotides with or without the deletion of additional adjacent nucleotides in the intron sequences of exon 53 to mediate its skipping. Although all of these strategies reframe the codon starting at exon 53, Del45-52 alters the spectrin-like repeat conformation of the dystrophin protein and this may have an impact on the protein’s activity (4, 22–24).

### 2.3. Cell lines

The myoblast cell lines AB1071 containing Del45-52 and KM1328 containing Del52 used in this study were obtained from Pr. Vincent Mouly’s Lab at the Myology Research Center of the Institute of Myology, 75013 Paris, France. These cells were prepared by Anne Bigot and Kamel Mamchaoui from the same institution.

### 2.4. Plasmid

The pCMV-PE2, pU6-pegRNA-GG-acceptor and pU6-tevopreq1-GG-acceptor plasmids were a gift from David Liu (AddGene plasmids #132775, #132777, and #174038). Cloning in these plasmids was done as described by Anzalone et al. (20). Oligonucleotides used for the construction of pegRNAs were purchased from IDT Inc. (New Jersey, United States).

### 2.5. Cell culture

Briefly, HEK293T cells were grown in DMEM-HG medium (Wisent Inc., Quebec, Canada) supplemented with 10% FBS (Wisent Inc.) and 1% penicillin–streptomycin (Wisent Inc.) at 37°C with 5% CO_2_ in a humidified incubator. The day before transfection, cells were detached from the flask with a Trypsin–EDTA solution (Sigma-Aldrich Canada Inc., Oakville, Canada) and counted. Detached cells were plated in a 24-well plate at a density of 60,000 cells per well with 1 mL of culture medium. On the day of transfection, the medium was replaced with 500 μL of fresh medium. Cells were transfected with 1 μg of total DNA (500 ng of each plasmid when co-transfection was required) with Lipofectamine 2000 (Invitrogen™ Inc., Carlsbad, United States) according to the manufacturer’s instruction. The medium was changed to 1 mL of fresh medium 24 h later and cells were maintained in incubation for 72 h before genomic DNA extraction.

The human myoblasts were grown in a home-made medium made of 4 volumes of DMEM-HG medium for 1 volume of medium 199 (Invitrogen™ Inc., Carlsbad, United States) supplemented with: Fetuin 25 μg/mL (Life Technologies, Carlsbad, USA), hEGF: 5 ng/mL (Life Technologies), bFGF: 0.5 ng/mL (Life Technologies), Insulin: 5 μg/mL (Sigma-Aldrich Canada Inc., 91077C-1G), Dex: 0.2 μg/mL (Sigma-Aldrich Canada Inc.). A total of 2 μg of plasmids (1 μg of pCMV-PE2 plasmid and 1 μg of pU6-GG-acceptor plasmid containing the pegRNA sequence and the sgRNA for PE3) were added to 100,000 human myoblasts and electroporated with the Neon Transfection System (program: 1,100 volts /20 ms /2 pulses). These electroporated cells were added to one well of a 24-well culture plate containing 500 μL of the home-made medium. The electroporation medium was changed with 1 mL fresh medium after 24 h and cells were detached with trypsin and harvested in 1 mL culture medium 48 h later. Half of the harvested cells were used for DNA extraction and the remaining volume was transferred to one well of a 6-well culture plate containing 2 mL of the home made medium. At 80% to 90% confluency, the medium was changed with 2 mL DMEM containing 1% FBS to induce myoblast fusion to form myotubes, which were harvested a few days later for western blot analysis of dystrophin.

### 2.6. Genomic DNA preparation and PCR amplification

HEK293T cells were detached from wells directly with up and down pipetting of the culture medium and transferred in 1.5 mL Eppendorf tubes. Human myoblasts were detached using Trypsin–EDTA solution (Sigma-Aldrich Canada Inc.) and collected in 1 mL of the original medium. HEK293T cells or human myoblasts were centrifuged for 5 min at 9000 RPM at room temperature. Cell pellets were washed once with 1 mL of PBS 1× and centrifuged again for 5 min at 9,000 RPM. Genomic DNA was prepared using the DirectPCR Lysis Reagent (Viagen Biotech Inc., Los Angeles, United States). Briefly, 50 μL of DirectPCR Lysis Reagent containing 0.5 μL of a proteinase K solution (20 mg/mL) was added to each cell pellet and incubated overnight at 56°C followed by another incubation at 85°C for 45 min and centrifugation at 13,000 RPM for 5 min. 1 μL of each genomic DNA preparation (supernatant) was used for the PCR reaction. PCR temperature cycling was performed as follows: 98°C: 30 s and 35 cycles of 98°C: 10 s, 60°C: 20 s, 72°C: 45 s for each primer set (Table 1). A final extension at 72°C for 5 min was also performed. We used Phusion™ High-Fidelity DNA polymerase from Thermo Scientific Inc. (Massachusetts, United States) for all PCR reactions. 5 μL of amplicons was electrophorized in 1X TBE buffer on 1% agarose gel to control the PCR reaction qualities and to make sure that only one specific band was present.

**Table 1:** Primer sequences.

Names	Primers sequences
PrAcceptor 51-Fwd	TTATTTCCCTGGCAAGGTCTG
PrAcceptor 51-Rev	CTAGGAGAGTAAAGTGATTGGTGG
PrSeq Acceptor 51	GTCCAGGCATGAGAATGAGC
Prdonor 51-Fwd	TTGATGTTGGAGGTACCTGCTC
Prdonor 51-Rev	TGCTGAGAGAGAAACAGTTGCC
PrSeq donor 51	CCTAAGAACTGGTGGGAAATGG
Prdonor 53-Fwd	GTCTCCTCCAGACTAGCATTTACT
Prdonor 53-Rev	ACTCCGTCCTCCTGACGAACTC
PrSeq donor 53	CAGAATCAGTGGGATGAAGTAC
Prdel52-RTPCR-Fwd	GATCTGAGCTCTGAGTGGAAGG
Prdel52-RTPCR-Rev	GCTTCCAGCCATTGTGTTGAATCC
Prdel45-52-RTPCR-Fwd	CAGTGGCTAACAGAAGCTGAACAG
Prdel45-52-RTPCR-Rev	ATCATGTGGACTTTTCTGGTATC
Pr deepSeq acceptor 51-Fwd	ACACTGACGACATGGTTCTACAGTCATGAATAAGAGTTTGGCTC
Pr deepSeq acceptor 51-Rev	TACGGTAGCAGAGACTTGGTCTGATCAAGCAGAGAAAGCCAGTC

### 2.7. Sanger sequencing

Amplicons from PCR (remaining 45 μL) were sent to the sequencing platform of the CHU de Québec Research Center (https://sequences.ulaval.ca/murin/servseq.pageaccueil) for Sanger sequencing. An internal primer (Table 1) was used for polymerisation using the BigDye™ Terminator v3.1. Sequences were analyzed with the EditR online program (https://moriaritylab.shinyapps.io/editr_v10/) (25) to look for editing efficacy for point mutations or TIDE (Tracking of Indels by Decomposition) software to check the editing percentage for insertion and deletions (https://tide.nki.nl/) (26).

### 2.8. Deep sequencing analysis

Deep sequencing samples were prepared following the PCR protocol described above with special primers containing a bar code sequence (BCS) for subsequent deep sequencing (Table 1). PCR samples of 300 bp amplicons’ length were sent to Genome Quebec Innovation Centre at McGill University (https://cesgq.com/en-services#en-sequencing) to sequence amplicons with the Illumina sequencer. Roughly 6,000–10,000 reads were obtained per sample. Illumina sequencing results were analyzed with the CRISPResso 2 online program (https://crispresso.pinellolab.partners.org/) according to the authors’ guidelines (27).

### 2.9. Western blot analysis

**Figure 3:**
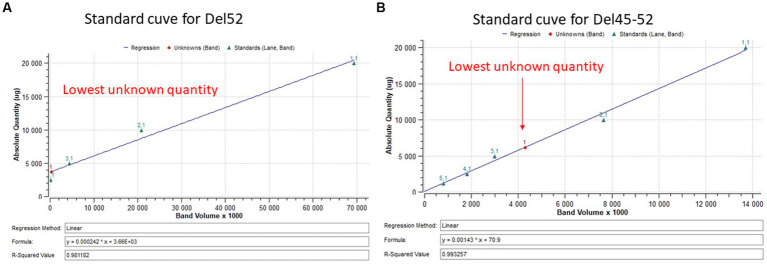
Standard curve for dystrophin quantification. **(A,B)** Represent the standard curve for the quantification of dystrophin restoration in Del52 and Del45-52. The figures indicate the linear curve with different ploted parameters.

Myotubes were detached directly from a culture plate with 400 μL of lysis buffer supplemented with protease inhibitors. Then, 1 μL of extracted proteins and different concentrations of BSA (used as standards) were placed on a nitrocellulose membrane and coloured with amido black 10B. The membrane was scanned by ChemiDoc XRS + system (Bio-Rad Laboratories Inc., Hercules, CA) and quantified using Image Lab 6.0.1 software (Bio-Rad Laboratories Inc.) according to the manufacturer’s instructions. 20 μg of extracted protein samples were separated by SDS-PAGE 4–7% (Bio-Rad Laboratories Inc.,) and transferred onto a Nitrocellulose membrane. A mouse monoclonal antibody against dystrophin (clone MANDYS8, ABNOVA Inc., Taipei, China) was used for an immunoblotting analysis. HRP conjugated goat anti-mouse (Thermo Scientific Inc.) was used as a secondary antibody. The membrane was developed using Clarity™ Western ECL substrate (Bio-Rad Laboratories Inc.) and scanned by ChemiDoc XRS + system (Bio-Rad Laboratories Inc.). The results were analyzed using the Bio Rad image lab 6.1 software (Bio-Rad Laboratories Inc.). The percentage of dystrophin was extrapolated in a standard curve made of normalized dystrophin quantity from 20 μg of total protein decreasing in a ratio of 2 (Figure 3).

### 2.10. RT-PCR analysis

RNAs from treated and untreated myoblasts containing Del52 or Del45-52 were isolated with TRIZOL according to the manufacturer’s protocol (Sigma-Aldrich Inc., St-Louis, MO, United States). cDNAs were then prepared using the high-capacity cDNA Reverse Transcription kits (Thermo Fisher Scientific Inc., MA, USA). Two pairs of primers (Table 1) were designed to amplify in exon 50 and 53 (for the Del52) or exon 44 and 54 (for Del45-52) were used PCR amplified the RT-PCR products. Two bands were expected in treated myoblasts that skipped either exon 51 (for the Del52) or exon 53 (For Del45-52).

### 2.11. Off-target prediction

The off-target prediction for the different Spacer sequences used where done using the CRISPOR software (28). The spacer sequence used for the modifications at the splice acceptor of exon 51 (ref. Table 2A) showed 15 off-targets prediction sites with 3 mismatches in the sequence. The spacer sequence used for the modifications at the splice donor of exon 51 (ref. Tables 2B-E) showed 1 and 5 off-targets sites with 2 and 3 mismatches, respectively. The spacer sequence used for the modifications at the position +6 of the splice donor of exon 53 (ref. Tables 2I,F,H) showed 3 and 33 off-targets sites with 2 and 3 mismatches, respectively. The spacer sequence used for the modifications at the position +1 of the splice donor of exon 53 (ref. Tables 2G,H) showed 2 and 12 off-targets sites with 2 and 3 mismatches, respectively.

**Table 2:** pegRNA sequences (nucleotides in small letters represent the intended edit).

Names	Spacer sequences	PBS sequences	RTT sequences	sgRNA sequences for PE3	sgRNA sequences for PE3b
**(A)** Substitution at the splice acceptor site of exon 51 (nucleotides in small letters are intended modification)					
PegRNA1	ACCAGAGTAACAGTCTGAGT	CAGACTGTTACTC	TTTaaCTCCTACT	TGACTTATTGTTATTGAAAT,AATGCCATCTTCCTTGATGT	GACTCCTACTCAGACTGTTACTC
PegRNA2	ACCAGAGTAACAGTCTGAGT	CAGACTGTTACTC	TATTTTaaCTCCTACT	TGACTTATTGTTATTGAAAT, AATGCCATCTTCCTTGATGT	GACTCCTACTCAGACTGTTACTC
PegRNA3	ACCAGAGTAACAGTCTGAGT	CAGACTGTTACTCTG	aaCTCCTACT	TGACTTATTGTTATTGAAAT, AATGCCATCTTCCTTGATGT	GACTCCTACTCAGACTGTTACTC
PegRNA4	ACCAGAGTAACAGTCTGAGT	CAGACTGTTACTCTG	TTTaaCTCCTACT	TGACTTATTGTTATTGAAAT, AATGCCATCTTCCTTGATGT	GACTCCTACTCAGACTGTTACTC
PegRNA5	ACCAGAGTAACAGTCTGAGT	CAGACTGTTACTCTG	TATTTTaaCTCCTACT	TGACTTATTGTTATTGAAAT, AATGCCATCTTCCTTGATGT	GACTCCTACTCAGACTGTTACTC
**(B)** Insertion of A at the end of exon 51					
PegRNA1	CGAGATGATCATCAAGCAGA	GCTTGATGATCATCTCGT	TTTCTCATACtCTTCT	CATCATTAAATTACAATCTA	
PegRNA2	CGAGATGATCATCAAGCAGA	GCTTGATGATCATCT	ATTTTTTCTCATACtCTTCT	CATCATTAAATTACAATCTA	
PegRNA3	CGAGATGATCATCAAGCAGA	GCTTGATGATCATCT	CCAACTTTTATCATTTTTTCTCATACtCTTCT	CATCATTAAATTACAATCTA	
PegRNA4	CGAGATGATCATCAAGCAGA	GCTTGATGATCATCT	TTTCTCATAtCaTTCT	CATCATTAAATTACAATCTA	
PegRNA5	CGAGATGATCATCAAGCAGA	GCTTGATGATCATCT	TTTCTCATAtCaTTCT	CATCATTAAATTACAATCTA	
PegRNA6	CGAGATGATCATCAAGCAGA	GCTTGATGATCATCT	TTTCTCATAtaaTTCT	CATCATTAAATTACAATCTA	
**(C)** pegRNAs optimization to increase the editing efficiency					
SubSpD51−1	CGAGATGATCATCAAGCAGA	GCTTGATGATCAT	GTGCTTTTCTCATtgCTTtT	CATCATTAAATTACAATCTA	
SubSpD51−2	CGAGATGATCATCAAGCAGA	GCTTGATGATCAT	GTGCTCTGCCAACTTTTATCATTTTTTCTCATtgCTTtTGC	CATCATTAAATTACAATCTA	
SubSpD51−3	CGAGATGATCATCAAGCAGA	GCTTGATGATCAT	GTGCAAAAAAGAAAAACTTCTGCCAACTTTTATCATTTTTTCTCATtgCTTtT	CATCATTAAATTACAATCTA	
SubSpD51−4	CGAGATGATCATCAAGCAGA	GCTTGATGATCAT	GTGCTTTTCTCATggCTTtT	CATCATTAAATTACAATCTA	
SubSpD51−5	CGAGATGATCATCAAGCAGA	GCTTGATGATCAT	GTGCTCTGCCAACTTTTATCATTTTTTCTCATggCTTtT	CATCATTAAATTACAATCTA	
SubSpD51−6	CGAGATGATCATCAAGCAGA	GCTTGATGATCAT	GTGCAAAAAAGAAAAACTTCTGCCAACTTTTATCATTTTTTCTCATggCTTtT	CATCATTAAATTACAATCTA	
SubSpD51−7	CGAGATGATCATCAAGCAGA	GCTTGATGATCAT	GTGCTTTTCTCATcgCTTtT	CATCATTAAATTACAATCTA	
SubSpD51−8	CGAGATGATCATCAAGCAGA	GCTTGATGATCAT	GTGCTCTGCCAACTTTTATCATTTTTTCTCATcgCTTtT	CATCATTAAATTACAATCTA	
SubSpD51−9	CGAGATGATCATCAAGCAGA	GCTTGATGATCAT	GTGCAAAAAAGAAAAACTTCTGCCAACTTTTATCATTTTTTCTCATcgCTTtT	CATCATTAAATTACAATCTA	
SubSpD51−10	CGAGATGATCATCAAGCAGA	GCTTGATGATCAT	TTTTCTCATcgCTTtT	CATCATTAAATTACAATCTA	
SubSpD51−11	CGAGATGATCATCAAGCAGA	GCTTGATGATCAT	TTTTCTCATcgtTTtT	CATCATTAAATTACAATCTA	
SubSpD51−12	CGAGATGATCATCAAGCAGA	GCTTGATGATCAT	TTTTCTCATggtTTtT	CATCATTAAATTACAATCTA	
SubSpD51−13	CGAGATGATCATCAAGCAGA	GCTTGATGATCAT	TTTTCTCATcaCTTtT	CATCATTAAATTACAATCTA	
SubSpD51−14	CGAGATGATCATCAAGCAGA	GCTTGATGATCAT	TTTTCTCATcatTTtT	CATCATTAAATTACAATCTA	
SubSpD51−15	CGAGATGATCATCAAGCAGA	GCTTGATGATCAT	CATcgCTTtT	CATCATTAAATTACAATCTA	
SubSpD51−16	CGAGATGATCATCAAGCAGA	GCTTGATGATCAT	CATggCTTtT	CATCATTAAATTACAATCTA	
**(D)** Insertion of nucleotides at the exon 51 splice donor site					
InsSpD51+6–1	CGAGATGATCATCAAGCAGA	GCTTGATGATCAT	TTTTCTCATAtCCTTCT	CATCATTAAATTACAATCTA	
InsSpD51+6–2	CGAGATGATCATCAAGCAGA	GCTTGATGATCAT	TTTTCTCATAgCCTTCT	CATCATTAAATTACAATCTA	
InsSpD51+6–3	CGAGATGATCATCAAGCAGA	GCTTGATGATCAT	TTTTCTCATAtgCCTTCT	CATCATTAAATTACAATCTA	
InsSpD51+6–4	CGAGATGATCATCAAGCAGA	GCTTGATGATCAT	TTTTCTCATAtgtCCTTCT	CATCATTAAATTACAATCTA	
InsSpD51+6–5	CGAGATGATCATCAAGCAGA	GCTTGATGATCAT	TTTTCTCATAgggCCTTCT	CATCATTAAATTACAATCTA	
InsSpD51+6–6	CGAGATGATCATCAAGCAGA	GCTTGATGATCAT	TTTTCTCATAtttCCTTCT	CATCATTAAATTACAATCTA	
**(E)** Deletion of nucleotides in exon 51 splice donor site					
DelSpD51+6–1	CGAGATGATCATCAAGCAGA	GCTTGATGATCAT	ATTTTTTCTCATCTTCT	CATCATTAAATTACAATCTA	
DelSpD51+6–2	CGAGATGATCATCAAGCAGA	GCTTGATGATCAT	TCATTTTTTCTCCTTCT	CATCATTAAATTACAATCTA	
DelSpD51+6–3	CGAGATGATCATCAAGCAGA	GCTTGATGATCAT	ATCATTTTTTCCTTCT	CATCATTAAATTACAATCTA	
**(F)** Substitution at +6 in the splice donor site of exon 53					
SubSpD53+6–1	AAAGAAAATCACAGAAACCA	TTTCTGTGATTTT	CTAggaTTGG	CATCATTAAATTACAATCTA	
SubSpD53+6–2	AAAGAAAATCACAGAAACCA	TTTCTGTGATTTT	ATACTAggaTTGG	CATCATTAAATTACAATCTA	
SubSpD53+6–3	AAAGAAAATCACAGAAACCA	TTTCTGTGATTTT	CTAcgaTTcG	CATCATTAAATTACAATCTA	
SubSpD53+6–4	AAAGAAAATCACAGAAACCA	TTTCTGTGATTTT	TTGATACTAggaTTGG	CATCATTAAATTACAATCTA	
SubSpD53+6–5	AAAGAAAATCACAGAAACCA	TTTCTGTGATTTT	TTGATACTAcgaTTGG	CATCATTAAATTACAATCTA	
SubSpD53+6–6	AAAGAAAATCACAGAAACCA	TTTCTGTGATTTT	TTGATACTAggaTTcG	CATCATTAAATTACAATCTA	
SubSpD53+6–7	AAAGAAAATCACAGAAACCA	TTTCTGTGATTTT	TTGATACTAcgaTTcG	CATCATTAAATTACAATCTA	
**(G)** Substitution at +1 in the splice donor site of exon 53					
SubSpD53+1–1	GGTATCTTTGATACTAACCT	TTAGTATCAAAGA	GAAACgAAac	CCAGAGCCAAGCTTGAGTCA	
SubSpD53+1–2	GGTATCTTTGATACTAACCT	TTAGTATCAAAGA	GAAACaAAac	CCAGAGCCAAGCTTGAGTCA	
SubSpD53+1–3	GGTATCTTTGATACTAACCT	TTAGTATCAAAGA	ACAGAAACgAAac	CCAGAGCCAAGCTTGAGTCA	
SubSpD53+1–4	GGTATCTTTGATACTAACCT	TTAGTATCAAAGA	ACAGAAACaAAac	CCAGAGCCAAGCTTGAGTCA	
SubSpD53+1–5	GGTATCTTTGATACTAACCT	TTAGTATCAAAGA	ATCACAGAAACgAAac	CCAGAGCCAAGCTTGAGTCA	
SubSpD53+1–6	GGTATCTTTGATACTAACCT	TTAGTATCAAAGA	ATCACAGAAACaAAac	CCAGAGCCAAGCTTGAGTCA	
**(H)** Insertion of nucleotides at the splice donor of exon 53					
InsSpD53+6–1	AAAGAAAATCACAGAAACCA	TTTCTGTGATTTTCT	ATACTAgggggaTTcG	CATCATTAAATTACAATCTA	
InsSpD53+6–2	AAAGAAAATCACAGAAACCA	TTTCTGTGATTTTCT	TTGATACTAgggggaTTcG	CATCATTAAATTACAATCTA	
InsSpD53+6–3	AAAGAAAATCACAGAAACCA	TTTCTGTGATTTTCT	TACTAggccgggaTTcG	CATCATTAAATTACAATCTA	
InsSpD53+6–4	AAAGAAAATCACAGAAACCA	TTTCTGTGATTTTCT	GATACTAggccgggaTTcG	CATCATTAAATTACAATCTA	
InsSpD53+1–1	GGTATCTTTGATACTAACCT	TTAGTATCAAAGA	ACgAAacccc	CCAGAGCCAAGCTTGAGTCA	
InsSpD53+1–2	GGTATCTTTGATACTAACCT	TTAGTATCAAAGATA	GAAACgAAacccc	CCAGAGCCAAGCTTGAGTCA	
InsSpD53+1–3	GGTATCTTTGATACTAACCT	TTAGTATCAAAGATA	ACAGAAACgAAacccc	CCAGAGCCAAGCTTGAGTCA	
InsSpD53+1–4	GGTATCTTTGATACTAACCT	TTAGTATCAAAGATA	AGAAACgAAaccggcc	CCAGAGCCAAGCTTGAGTCA	
**(I)** Deletion at +6 of nucleotides at the splice donor of exon 53					
DelSpD53+6–1	AAAGAAAATCACAGAAACCA	TTTCTGTGATTTTCT	TTGATACTAaTTGG	CATCATTAAATTACAATCTA	
DelSpD53+6–2	AAAGAAAATCACAGAAACCA	TTTCTGTGATTTTCT	ctTTGATACTAaGG	CATCATTAAATTACAATCTA	
DelSpD53+6–3	AAAGAAAATCACAGAAACCA	TTTCTGTGATTTTCT	atctttgatactGG	CATCATTAAATTACAATCTA	
DelSpD53+6–4	AAAGAAAATCACAGAAACCA	TTTCTGTGATTTTCT	TTGATACTACTTGG	CATCATTAAATTACAATCTA	
DelSpD53+1–1	AAAGAAAATCACAGAAACCA	TTAGTATCAAAGATA	ATCACAGAAACaAAa	CCAGAGCCAAGCTTGAGTCA	

### 2.12. Statistical analysis

The data was analyzed using the Graph Pad PRISM 5.0 software package (Graph Pad Software Inc., La Jolla, California, United States). The comparison between the mean editing percentage of single pegRNAs was performed using the Kruskal Wallis and the Bonferroni post test. A value of *p* < 0.05 was considered statistically significant for a 5% confidence interval.

## 3. Results

### 3.1. Correcting the frameshift in *DMD* Del52

#### 3.1.1. Modifying the splice acceptor site

**Figure 4:**
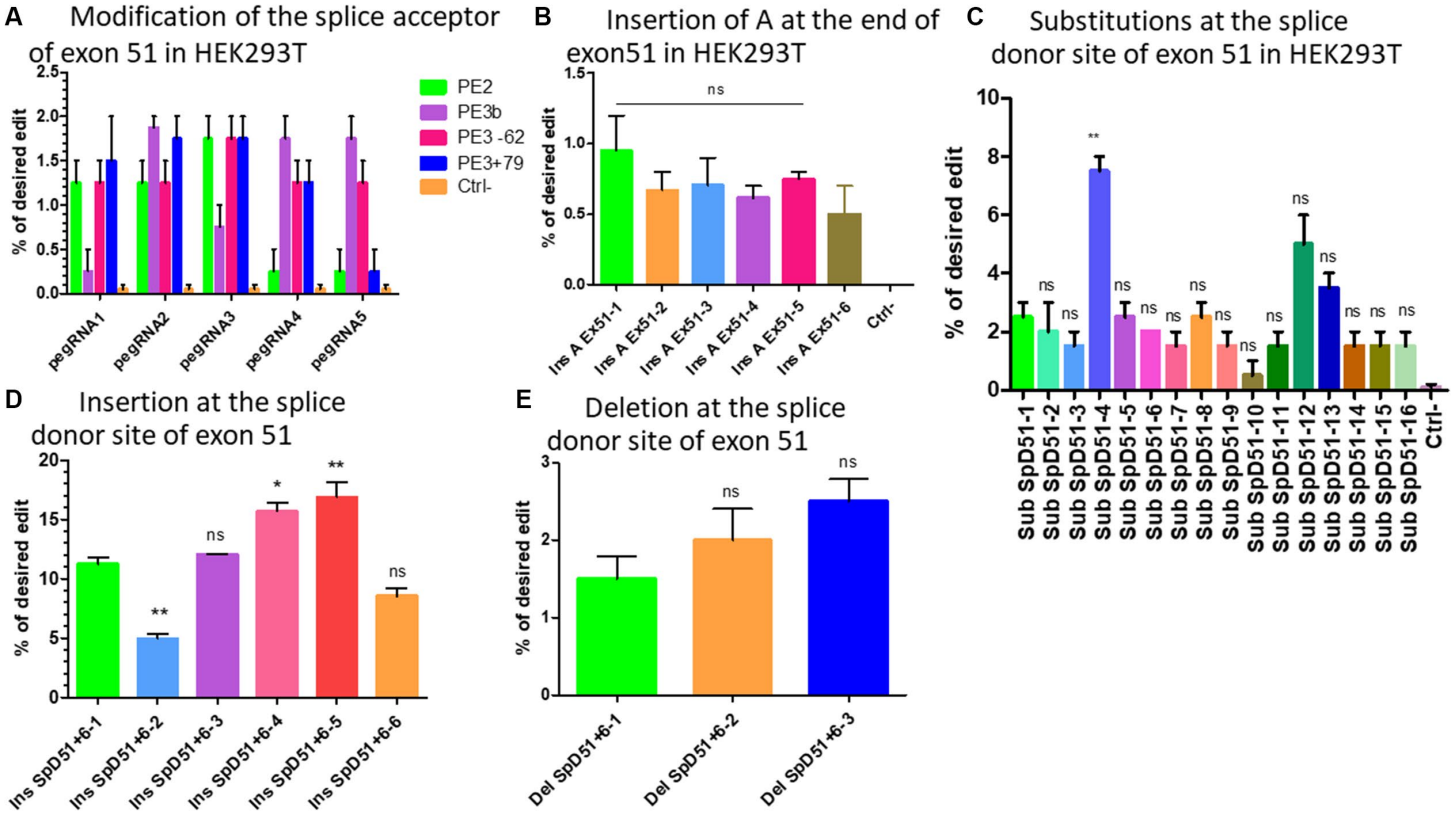
The modification strategies for the skipping or reframing of exon 51. **(A)** Represents the substitution Prime editing results at the splice acceptor site of exon 51 combining PE2 (in green), PE3 (in pink for the nick at the position −62 and in blue for the nick at the position +79), PE3b (in purple) strategies in HEK293T cells. **(B)** Represents the Prime editing insertion strategy at the end of exon 51 to restore the reading frame in HEK293T cells (Ins A Ex51 is the pegRNA sequence for the insertion of A nucleotide at the end of exon 51). There was no significant difference (ns) between the different pegRNAs. **(C)** Shows the Prime editing results for the strategy consisting of changing one or the two nucleotides of the splice donor site of exon 51. Sub SpD51 is the pegRNA sequence for the substitution (Sub) of the splice donor 51 (SpD51). The editing percentage of pegRNAs were compare with the SubSpD51-1 pegRNA using the Kruskal Wallis test. ** indicates a value of *p* < 0.05 and ns indicates a nonsignificant difference. **(D)** Represents the results for the nucleotide insertions at the splice donor of exon 51 using pegRNA to edit at the position +6. **(E)** Represents the editing rate for the deletion of nucleotides at the splice donor site of exon 51. Ins SpD51 is the pegRNA sequence for the insertion (Ins) or deletion (Del) of the splice donor 51 (SpD51) at the position +6. Experiments were done in independent triplicates in HEK 293 T cells. ns indicates a nonsignificant difference and *, ** indicate the level of significance comparing different pegRNAs editing percentage using the Kruskal Wallis and the Bonferroni post-test.

We initially designed five different pegRNAs (Table 2A) to modify the splice acceptor site (AG) of exon 51 changing AG to AA at the position +9 (9 nucleotides 3′ from the nick site induced by SpCas9n). In addition, we used two different sgRNAs to induce a nick at +79 or −62 for the PE3 strategy (which uses an additional sgRNA to nick the non-edited DNA strand at the distance of 40 to 150 nucleotides from the pegRNA nick site) (20). Another sgRNA was used for the PE3b approach, which uses an additional sgRNA containing the intended modification to nick the non-edited DNA strand at the modification site. The binding of this sequence is possible only if the pegRNA induced the intended mutation (20). We did not change the PAM sequence in the RTT sequences because this would have changed the amino acid encoded by this part of the *DMD* gene. The Illumina deep sequencing results indicated 0.5% to 1.8% modifications on average with different pegRNAs (Figure 4A). There were no detectable INDELs. These weak results might be due to the fact that this approach did not involve mutating the PAM. Mutating the PAM during Prime editing has been shown to increase Prime editing efficiency (21).

#### 3.1.2. Inserting an adenine at the end of exon 51

Six different pegRNAs (Table 2B) were designed to add an Adenine at position +5 at the end of exon 51. A second sgRNA was designed to induce a nick at +69 for the PE3 approach (20). The deep sequencing results indicated up to 1% insertion of Adenine at the target (Figure 4B). There were no detectable INDELs. With such low levels of modification for the target site, various methods to improve the editing efficiency were investigated.

#### 3.1.3. Pegrna optimizations to modify the splice donor site of exon 51

We designed 16 optimized engineered pegRNAs (epegRNAs) (29) (Table 2C). These epegRNAs carried an additional pseudoknot extension sequence at their 3′ end that prevented the pegRNA from being degraded by exonucleases. This has been shown to significantly increase the Prime editing efficiency (29). In addition to mutating the PAM sequence and PE3 approach, one or two additional synonymous mutations were also added on either side of the intended modification to increase the editing efficiency (21, 30). Our group has recently shown that the addition of synonymous mutations has the potential to increase the Prime editing efficacy in the *DMD* gene by 9.6-fold (21). These epegRNAs were configured to change the GT of the splice donor site of exon 51 to either CA (SubSpD51−1,2, and 3), CC (SubSpD51−4,5,6,12, and 16), CG (SubSpD51−7,8,9,10,11, and 15), or TG (SubSpD51−13 and 14). These four modifications of the epegRNAs were tested because we previously demonstrated that the nucleotide interconversions at the editing site significantly changed the editing outcome (21). The Sanger sequencing results showed up to 3%, 7.5%, 2.5%, and 1.5% edition for the four conversions, respectively. Changing GT to CC appears to be the best candidate (Figure 4C).

#### 3.1.4. Altering the splice donor site of exon 51 by inserting nucleotides between the GT nucleotides

Another strategy to mediate the skipping of exon 51 is to insert one or several nucleotides between the two nucleotides (GT) of the donor splice site to make it unavailable for the binding of the spliceosome proteins (31). We designed six epegRNAs (Table 2D) to mediate the insertion of either A, C, CA, ACA, CCC, or AAA at the position +6 from the nick site. This resulted in mutation rates of 11%, 4%, 12%, 16%, 17%, and 8%, respectively, for the above modifications. The insertion of CCC represented the best candidate for this strategy (Figure 4D).

#### 3.1.5. Altering the splice site of exon 51 by deleting nucleotides

As in the nucleotide insertion strategy, deleting the nucleotides of the splice sites of exon 51 will favor its skipping and link exon 50 to exon 53. We designed 3 new epegRNAs to induce the deletion of either GT (the splice site), GT**AT** (AT belonging to the intron sequence) or GT**ATGA** (ATGA belonging to the intron sequence) at the position +6 from the nick site (Table 2E). Results indicated deletions of 1.5%, 2%, and 2.5% of two (GT), four (GTAT), and six (GTATGA) nucleotides, respectively (Figure 4E). The editing rate increased with the number of nucleotides to be deleted.

#### 3.1.6. Restoring the dystrophin expression in Del52 patient myoblasts

**Figure 5:**
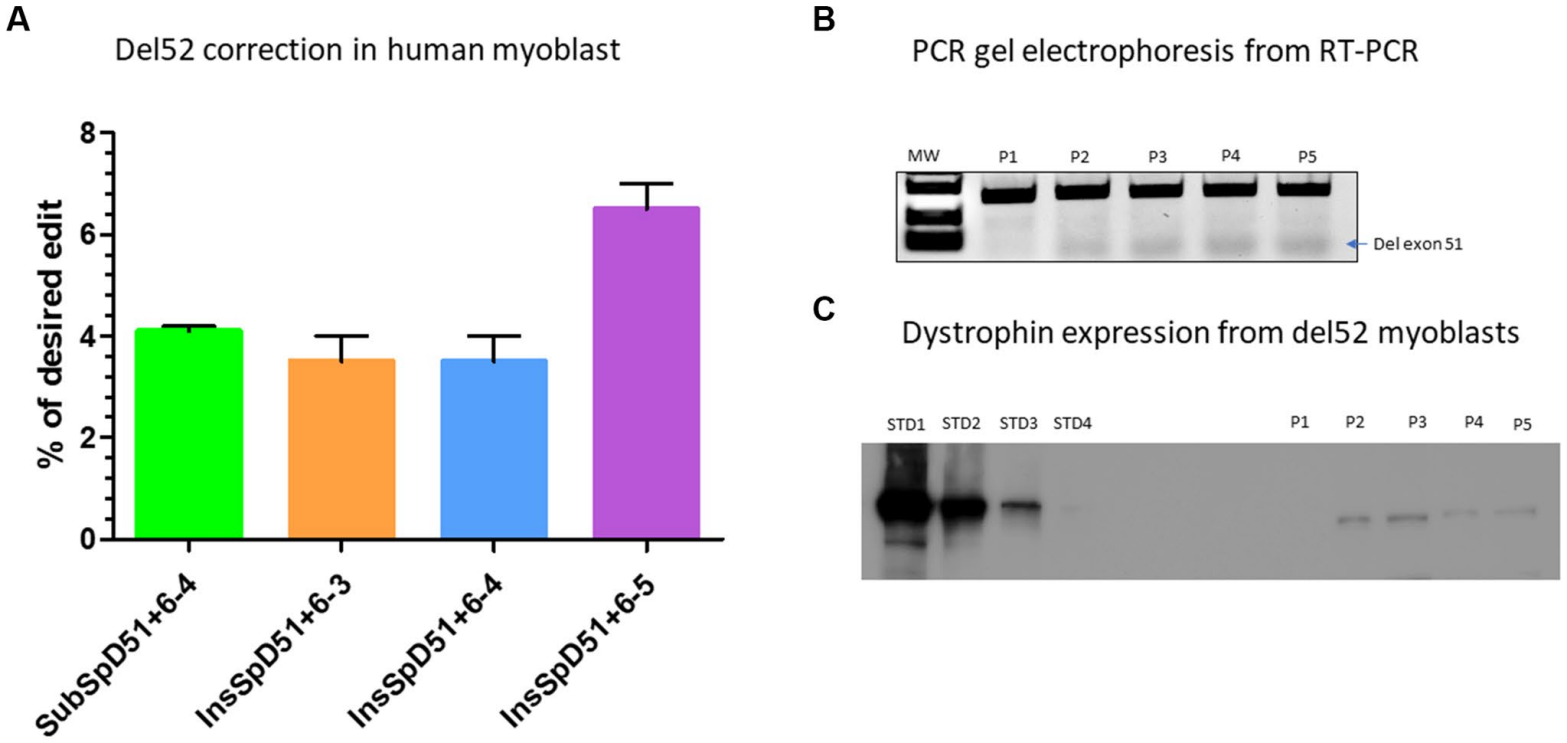
Correction of Del52 mutation in myoblasts. **(A)** Represents the Prime editing results of the correction of patient myoblasts carrying Del52 mutation using selected pegRNAs from previous experiments in HEK293T cells. **(B)** Represents the gel electrophoresis results from the PCR of RT-PCR samples. P1, P2, P3, P4, and P5 represent, respectively, the negative control (untreated Del52 patient myoblasts), SubSpD51+6–4, InsSpD51+6–3, InsSpD51+6–4, and InsSpD51+6–5. The lower size bands confirm the exon 51 skipping. **(C)** Represents the western blot result for the correction of Del52 in patient myoblasts. STD1, STD2, STD3, and STD4 represent the positive control standard bands(untreated healthy myoblasts), P1, P2, P3, P4, and P5 represent, respectively, the negative control (untreated Del52 patient myoblast), SubSpD51+6–4, InsSpD51+6–3, InsSpD51+6–4, and InsSpD51+6–5.

We selected four epegRNAs including the *SubSpD51*−*4* which achieved 7.5% modification of GT to CC at the splice donor site of exon 51 (Figure 4C), the *InsSpD51+6–3,* the *InsSpD51+6–4*, and *InsSpD51+6–5* which achieved 12%, 16%, and 17% insertion of CA, ACA and CCC between the GT splice donor site of exon 51, respectively (Figure 4D). These epegRNAs were used for the correction of DMD patient myoblasts carrying Del52. We performed an RT-PCR amplification (to verify the exon 51 skipping) and a western blot (to verify whether the dystrophin expression was restored). The Sanger results showed 4%, 3%, 3%, and 6.5% skipping of exon 51 with the *SubSpD51+6–4, InsSpD51+6–3, InsSpD51+6–4,* and *InsSpD51+6–5* epegRNAs, respectively (Figure 5A). The bands from RT-PCR products confirmed the skipping of exon 51 (corresponding to the lower band in Figure 5B). The Western blot carried out with 20 μg of total proteins confirmed a dystrophin expression of 9.9%, 9%, 9.3%, and 10% compared to the normal human myoblast expressing dystrophin (Figure 5C).

### 3.2. Correcting the reading frame in DMD Del45-52

#### 3.2.1. Modification of the splice donor site of exon 53

**Figure 6:**
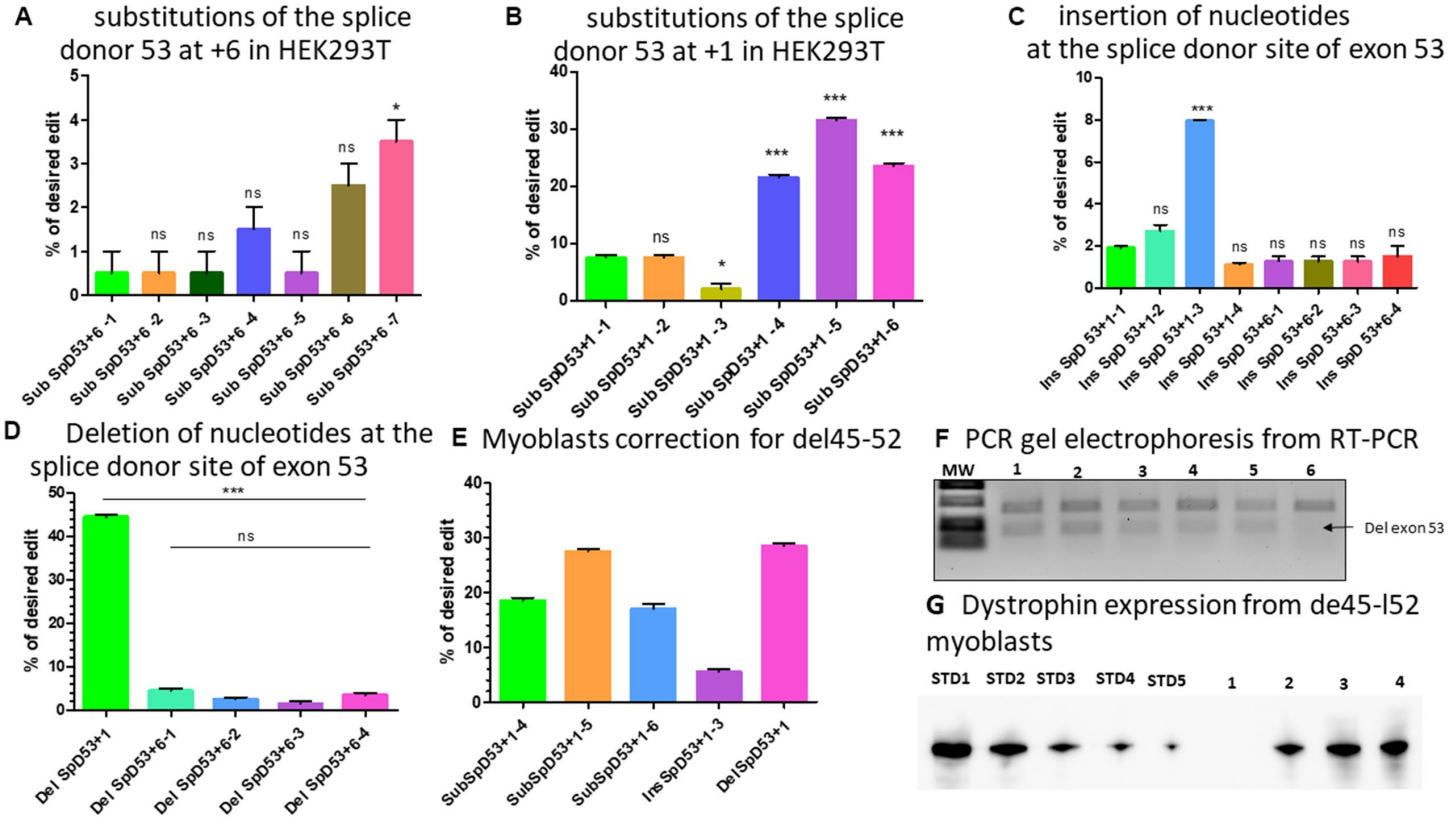
modifications at the splice site of exon 53. **(A)** Represents the editing percentage of Prime editing substitution at the position +6 of the splice donor 53. **(B)** Represents the editing results of Prime editing substitution at the position +1 of the splice donor 53. Experiments were done in independent triplicates in HEK 293 T cells. **(C)** Represents the Prime editing results for insertion of nucleotides at the splice donor site of exon 53. **(D)** Shows the Prime editing percentage for the deletion of nucleotides at the splice donor site of exon 53. Experiments were done in independent triplicates in HEK 293 T cells. **(E)** Represents the editing percentages of Del45-52 myoblasts treated with different epegRNAs. **(F)** Represents the gel electrophoresis results from the PCR of RT-PCR samples. 1, 2, 3, 4, 5, and 6 represent, respectively, the SubSpD53+1–4, SubSpD53+1–5, SubSpD53+1–6, InsSpD53+1–3, DelSpD53+1–1, and the negative control (untreated Del45-52 patient myoblasts). The lower size bands confirm the exon 53 skipping. **(G)** Represents the dystrophin expression detected by western blot after the correction of Del45-52 patient myoblasts. STD1, STD2, STD3, STD4, and STD5 correspond to positive control standard bands (untreated healthy myoblasts), and 1, 2, 3, and 4 correspond, respectively, to the negative control (untreated Del45-52 patient myoblast), SubSpD53+1–4, SubSpD53+1–5 and DelSpD53+1–1. The editing percentage of between pegRNAs were compare using the Kruskal Wallis test and the Bonferroni post-test. *, ** and *** indicate the level of significance for value of *p* < 0.05. ns indicates a nonsignificant difference.

There were two PAM sequences located near the splice donor site of exon 53 which could be used to modify the position +6 for Del52 or position +1 from the nick site depending on spacer sequence used for the epegRNAs (Tables 2F,G). We first designed different optimized epegRNAs which contained additional mutations at different positions around the target and a mutation for the PAM sequence. The epegRNAs were used in parallel with an additional sgRNA to perform the PE3 technique. This changed the GT to either CC or CG (Table 2F). As expected, results showed up to 2.5% CC and 3.5% CG modifications confirming the hypothesis that the position of the targeted modification plays a critical role in the editing outcome (Figure 6A). We next designed 6 other epegRNAs with almost the same configuration to mediate the edit at the position +1 (Table 2G). In these new sequences, the T of the GT splice site could not be included in the RTT sequence because the nick was between the GT nucleotides. We mutated the PAM sequence and added other synonymous mutations at positions +2 and +5. This configuration resulted in the conversion of up to 32% of G to C at the target (position +1) as well as the simultaneous conversion of G to A and C to G at the positions +2 and +5, respectively (Figure 6B).

#### 3.2.2. Insertion of nucleotides at the splice site

As shown with Del52, the insertion of three nucleotides (GGG) between GT of the exon 51 splice site at position +6 resulted in an editing rate of up to 17% (Figure 4D). We reasoned that increasing the number of nucleotides inserted might further improve the efficacy at the same position. We thus designed two additional epegRNAs to insert six nucleotides and two other epegRNAs to insert eight nucleotides at the position +6 (Table 2H). Unfortunately, we only observed up to 2% editing (Figure 6C). Increasing the length of the fragment to be inserted to either six or eight nucleotides had no positive impact on the outcome. We also designed three epegRNAs with different RTT lengths (RTT7, RTT10 and RTT13) to insert CCC at the position +1. This implied that the insertion was done at the beginning of the RTT sequence. An additional epegRNA (RTT13) was further designed to insert CGGCC at the same position. The results showed 2%, 2.7%, 8%, and 1.1% insertion of CCC and CGGCC, respectively (Figure 6C). The insertion of GGG or CCC between the nucleotides of the splice donor site to mediate exon skipping was the best formulation. The editing efficacy was influenced by the distance between the insertion site and the nick site.

#### 3.2.3. Deleting nucleotides at the splice donor site of exon 53

We designed four epegRNAs to mediate the deletion of two, four, or six nucleotides at the position +6 as for the Del52 mutation (Table 2I). As expected, these resulted in modifications of up to 4% (Figure 6D). The deletion strategy might not be appropriate at the position +6. As the epegRNA sequences that were designed to induce a modification at position +1 could not include the T of the GT splice site, we designed one optimized epegRNA to induce the deletion of the G at the position +1. Results indicated a 44% deletion of G (Figure 6D). These results suggested that the deletion strategies might be more appropriate for nucleotides close to the nick site.

#### 3.2.4. Restoring the dystrophin expression in *DMD* Del45-52 patient myoblasts

We selected five epegRNAs: *SubSpD53*+*1–4*, *SubSpD53*+*1–5,* and *SubSpD53*+*1–6,* achieved 20%, 32% and 22% substitutions of GT to CC and CG, respectively (Figure 6B), *InsSpD53*+*1–3* achieved 8% insertion of CCC between the GT splice donor site of exon 53 (Figure 6C), and the *DelSpD53*+*1,* achieved 44% deletion of G at the GT splice donor site of exon 53. These five epegRNAs were used to correct patient myoblasts carrying Del45-52. The Sanger sequencing results showed up to 28%, 6%, and 29% editing efficacy for substitutions, insertions, and deletions, respectively (Figure 6E). The RT-PCR demonstrated lower size bands (due to the skipping of exon 53) as expected for treated cells (Figure 6F). The Western blot carried out with 20 μg of total protein confirmed the dystrophin expression of 30.7%, 50.2%, and 50.9%, respectively, for *SubSpD53*+*1–4*, *SubSpD53*+*1–5* and *DelSpD53*+*1 pegRNAs* (Figure 6G) compared to the positive control.

## 4. Discussion

Since the initial development of Prime editing (20), many strategies have progressively been developed to significantly improve its efficacy. These include the modification in the PAM sequence combined to PE3 approach (20, 32), the addition of synonymous mutations in the RTT sequence (21), the protection of pegRNAs from exonucleases using pseudoknots at the 3′ extension (resulting in epegRNA) (29), the modification of the secondary structure of pegRNA (30), the modification of the Cas9n to make it more active and specific (33), the reduction of RT length (34, 35), the modification of the mismatch repair proteins (33) and the recruitment of transcription factors (36). In the current manuscript, we combined the pseudoknot strategy (epegRNA), the mutation in the PAM, the addition of synonymous mutations, and the PE3 to test different strategies for the correction of Del52 and Del45-52 mutations in DMD patient myoblasts.

Using optimized epegRNAs, we demonstrated that Prime editing can be used to substitute nucleotides at the splice donor sites of exons 51 and 53. The efficacy of this substitution depended on the position of the edit from the nick site and the type of nucleotides to be changed. We observed that the nucleotide interconversion to C and G was associated with a higher editing rate on average. The editing efficacy could be further improved by mutating additional nucleotides directly next to the target. We believe that the proximity of the mutations might highly disfavour the mismatch repair mechanism that has been shown to reduce the Prime editing efficacy (33, 37, 38). The deletion strategy seemed to be more efficient when the edit was done close to the nick site. That was not the case with the insertion strategies which prefer not to be so close to the nick site. On average, the insertion of three nucleotides (preferably GGG or CCC) was associated with a high target mutation rate. Many of the optimized epegRNAs designed for the different strategies were not efficient enough to induce the intended modification probably because of the complex mechanism of mRNA splicing at the splice sites (39).

Other nickase tools such as base editing have already been used by other groups for the correction of point mutations (in exon 20, 23 and 53), Del48-54, and Del51 in the *DMD* gene (13–16). The results were higher on average than those obtained by Prime editing. However, these studies also showed high levels of undesired conversions in the editing windows which remains one of the most challenging issues with base editors. Thus, Prime editing has revealed itself to be more precise (less than 1% INDELs) at modifying the *DMD* gene. However, it has been noted that a fragment of the scaffold sequence of pegRNA could be inserted in the target during the reverse transcription of the RTT (40). This effect was not observed in the current study. The results obtained in the present study might be significantly improved when combined with the most recent optimization strategies mentioned above like the inhibition of mismatch repair genes, the reduction of RT length or the recruitment of more transcription factors.

The editing percentage obtain in the present study is very encouraging for a potential *in vivo* translation. The major anticipated limitation will be the delivery system. The PE length is about 6.3 kb and cannot be package in a single vector. An avenue might be the delivery of PE protein using VLPs (41). However, a marginal level of 3%–15% dystrophin expression has been shown to improve the phenotype of DMD mice and human (42–44). This is because a muscle fibre contains more than a thousand nuclei and a dystrophin nuclear domain is made of about 30 nuclei (45).

In this study, substitution, deletion, and insertion strategies were used at the splice sites of exons 51 and 53 of the *DMD* gene to correct the frameshift in *DMD* Del52 and Del45-52. The highlight of this strategy is that it might benefit the large majority of DMD patients having mutations in the region spanning exons 45 to 52, which is one of the two important mutational hotspots in DMD (4, 5). This strategy might be further adapted to develop a multiplex therapeutic approach for DMD.

## Data availability statement

The datasets presented in this study can be found in online repositories. The names of the repository/repositories and accession number(s) can be found below: BioProject PRJNA922596.

## Author contributions

CM conceived and designed the experiments. CM, JeR, YL, JoR, and PY performed the experiments. VM, AB, and KM prepared the myoblast cell lines. GL proofread the manuscript. JT supervised the research. CM and JT wrote the manuscript. All authors contributed to the article and approved the submitted version.

## Funding

This work was supported by grants from Jesse’s Journey the Foundation for Cell and Gene Therapy, the Canadian Institute of Health Research (CIHR) and the TheCell network (Réseau de Thérapie Cellulaire, Tissulaire et Génique du Québec) of the Fond de Recherche du Québec en Santé (FRQS).

## Conflict of interest

The authors declare that the research was conducted in the absence of any commercial or financial relationships that could be construed as a potential conflict of interest.

## Publisher’s note

All claims expressed in this article are solely those of the authors and do not necessarily represent those of their affiliated organizations, or those of the publisher, the editors and the reviewers. Any product that may be evaluated in this article, or claim that may be made by its manufacturer, is not guaranteed or endorsed by the publisher.
